# Histological evaluation of nonsurgical periodontal treatment with and without the use of sodium hypochlorite / amino acids and cross-linked hyaluronic acid gels in dogs

**DOI:** 10.1007/s00784-024-05674-7

**Published:** 2024-04-27

**Authors:** Yoshinori Shirakata, Toshiaki Nakamura, Fumiaki Setoguchi, Takatomo Imafuji, Yukiya Shinohara, Shohei Matsumura, Masayuki Iwata, Kazuyuki Noguchi, Eglė Ramanauskaite, Anton Sculean

**Affiliations:** 1 Department of Periodontology, Kagoshima University Graduate School of Medical and Dental Sciences, Kagoshima, Japan; 2 Clinic of Dental and Oral Pathology, Lithuanian University of Health Sciences, Kaunas, Lithuania; 3 Department of Periodontology, University of Bern, Bern, Switzerland

**Keywords:** Periodontitis, Periodontal wound healing/regeneration, Non-surgical periodontal therapy, Cross-linked hyaluronic acid, Sodium hypochlorite/amino acids

## Abstract

**Objectives:**

To evaluate periodontal wound healing following scaling and root planing (SRP) in conjunction with the application of sodium hypochlorite/amino acids and cross-linked hyaluronic acid (xHyA) gels in dogs.

**Materials and Methods:**

In four beagle dogs, 2-wall intrabony defects were created and metal strips were placed around the teeth. Clinical parameters were measured 4 weeks after plaque accumulation. The experimental root surfaces were subjected to SRP with either the subgingival application of a sodium hypochlorite/amino acid gel and a xHyA gel (test group) or SRP alone (control group) using a split-mouth design. Clinical parameters were re-evaluated at 6 weeks. The animals were sacrificed at 8 weeks for histological analysis.

**Results:**

The test group showed significant improvements in all clinical parameters compared to the control group. Histologically, the test group exhibited statistically significantly greater new bone formation [i.e., length of newly formed bone, new bone area] compared with the control group (*p* < 0.05). Furthermore, statistically significantly greater formation of new attachment [i.e., linear length of new cementum adjacently to newly formed bone with inserting collagen fibers] and new cementum was detected in the test group compared with the control group at 8 weeks (*p* < 0.05 and *p* < 0.01, respectively).

**Conclusion:**

The adjunctive subgingival application of sodium hypochlorite/amino acid and xHyA gels to SRP offers an innovative novel approach to enhance periodontal wound healing/regeneration.

**Clinical relevance:**

The present findings have for the first-time shown histologic evidence for periodontal regeneration in support of this novel treatment modality.

## Introduction

Periodontitis is a highly prevalent chronic inflammatory disease caused by dysbiotic dental plaque, leading to the destruction of connective tissue attachment and loss of alveolar bone, ultimately resulting in tooth loss [1–3]. The treatment of periodontitis is multi-staged and first steps of non-surgical periodontal therapy (NSPT) include supragingival plaque control, followed by subgingival scaling and root planing (SRP), aimed at eliminating biofilms, endotoxins, and calculus. This treatment reduces inflammation and reestablishes a favorable environment for oral hygiene measures [4–6]. The efficacy of SRP has been well reported by gains in clinical attachment level (CAL), reductions in periodontal pocket depth (PPD) and in the frequency of bleeding on probing (BOP) [4, 7, 8]. However, SRP does not always result in closure of periodontal pockets and the outcomes may be influenced by several patient related factors (e.g., smoking level, oral hygiene), anatomical factors (e.g., tooth type and surface, furcation involvement) and operator’s experience [9–11]. The available evidence from histological studies indicates that SRP typically leads to a reparative type of healing characterized by formation of a long junctional epithelium and limited or no regeneration of cementum, periodontal ligament and bone [12–16]. Consequently, various strategies including the use of antibiotics, antiseptics and different biological agents adjunctive to SRP have been used to effectively control the bacterial biofilm caused inflammation and enhance wound healing [15, 17–25].

Recently, a novel formulation of sodium hypochlorite (NaOCL) gel buffered with leucine, lysine, and glutamine acid (Perisolv®, Regedent AG, Zürich, Switzerland) has been suggested as an adjunct to SRP. Since it has been shown that the active ingredients in the gel create chloramines, which have a strong antimicrobial effect and can penetrate the biofilm [26], it has been suggested that its use may aid for both the mechanical removal of hard and soft subgingival bacterial deposits and the detoxification of the root surface [26, 27]. In this respect, positive clinical effects of a sodium hypochlorite gel were reported in studies treating deep pockets at teeth [28, 29] and dental implants [27].

Hyaluronic acid (HyA) is a major natural glycosaminoglycan component of the extracellular matrix in many tissues such as skin, joints, eyes, and periodontium and has several unique physiochemical and biological properties including hygroscopic, viscoelastic, bacteriostatic, anti-inflammatory, anti-oedematous, pro-angiogenetic and osteoinductive nature [30–35]. HyA is currently also available in cross-linked form (cross-linked HyA: xHyA) for various applications in tissue engineering, serving as biologics/scaffolds to further improve the overall mechanical properties and provide a longer degradation period compared with non-cross-linked HyA [36, 37]. Results from clinical studies, indicate positive outcomes evidenced by significant gain of CAL, PPD reduction and improved BOP values have been reported following the adjunctive application of xHyA to nonsurgical and surgical periodontal therapy [17, 31, 38]. Furthermore, a recent series of preclinical studies has demonstrated periodontal regeneration, evidenced by formation of cementum, periodontal ligament, and bone, following the application of xHyA in conjunction with reconstructive periodontal surgery for recessions, intrabony, and furcation defects [39–41].

Very recently, a novel two-step approach consisting of enhanced biofilm removal during nonsurgical therapy by means of a sodium hypochlorite/amino acid followed by application of a xHyA gel was suggested to improve the outcomes of nonsurgical periodontal therapy [42–44]. Results from two case series have shown statistically significant clinical improvements compared to baseline following SRP in conjunction with sodium hypochlorite/amino acid and xHyA [42, 43], thus suggesting that this strategy may represent a valuable novel strategy in non-surgical periodontal treatment. Additionally, a randomized controlled clinical study has assessed the clinical outcomes achieved through either mechanical subgingival debridement in conjunction with a sodium hypochlorite/amino acids-containing gel followed by subsequent application of a xHyA gel, or mechanical debridement alone [44]. The results have shown that both treatments led to statistically significant improvements in all evaluated clinical parameters, but the adjunctive subgingival application of sodium hypochlorite/amino acids and xHyA to SRP resulted in statistically significantly greater improvements compared to SRP alone [44].

However, to the best of our knowledge, at present no histological data are available evaluating the healing following the use of this novel approach for non-surgical therapy. Therefore, the aim of this study was to histologically evaluate in dogs, the healing following nonsurgical periodontal therapy and in conjunction with sodium hypochlorite/amino acid gel and xHyA application.

## Methods and materials

### Animals

Four healthy male beagle dogs, 26 to 38 months of age and weighing 9 to 15 kg, were used in this study. The animals were housed and monitored daily for the duration of the study in the Animal Experimentation Facility Shin Nippon Biomedical Laboratories, Ltd., Kagoshima, Japan. They were kept in individual cages at 20–26 ℃, relative humidity of 30–70%, and a 12-h light/dark cycle. Approximately 300 g of solid food (NVE-10, Nippon Pet Food, Co., Ltd. Tokyo, Japan) was provided to each animal daily and water was available ad libitum. All procedures during the in-life phase were approved by the ethical committee of the Animal Research Center of Kagoshima University, Japan (Project Approval No. D22017; Date of approval: 23 January 2023). This study conformed to the ARRIVE guidelines for preclinical animal studies.

### Induction of experimental periodontitis

**Fig. 1:**
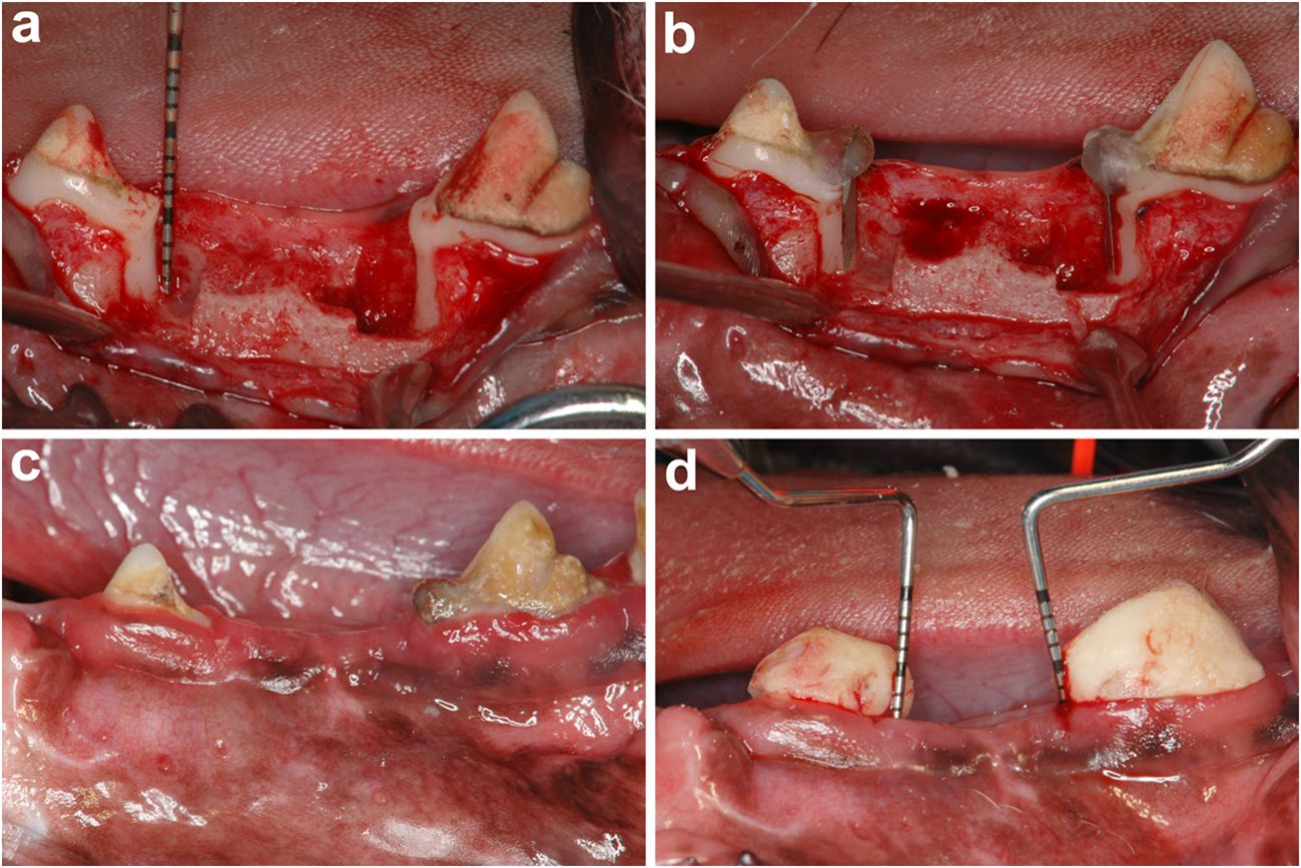
**a** Surgically created two-wall intrabony defects in mandible. **b** Placement of the metal strips on the denuded root surfaces. **c** Plaque accumulation after 4 weeks. **d** Standardized measurement of clinical parameters by using customized acrylic stents with guiding grooves at baseline

All surgical procedures were performed under general and local anesthesia using aseptic routines by one experienced surgeon (Yo.S.). Before surgical procedures, antibiotics (dihydrostreptomycin sulfate aqueous suspension for injection, 0.05 ml/kg; Mycillin Sol Meiji for veterinary use, Meiji Seika Pharma Co. Ltd, Tokyo, Japan) were administered intramuscularly. General anesthesia was induced with intramuscular injection using medetomidine hydrochloride (Domitor®, 0.08 ml/kg IM; Orion Corporation, Espoo, Finland), 0.08 ml/kg of midazolam (Dormicum®, IM; Maruichi Pharmaceutical, Osaka, Japan) and 0.02 ml/kg of butorphanol tartrate (Vetorphale® 5 mg, Meiji Seika Pharma, Tokyo, Japan). After sedation, the anesthesia was maintained by inhalation of sevoflurane (0.5%**–**5.0%, Mylan Pharma Co., Ltd. Osaka, Japan) and a nitrogen/oxygen (2:1) mixture using an intracircuit vaporizer for spontaneous breathing. Local anesthesia was achieved with lidocaine HCl/epinephrine (2%, 1:80,000; Xylocaine; Fujisawa Inc., Osaka, Japan). The bilateral mandibular first and third premolars were carefully extracted to provide enough space for creation of intrabony defects. After a 8-week healing interval, two-wall intrabony defects (5 mm wide and 5 mm deep) were prepared bilaterally at the mesial aspect of the mandibular fourth premolars (P4) and at the distal aspect of the mandibular second premolars (P2) (four defects per dog). Following elevation of the mucoperiosteal flap, defects were created by using fissure burs with a sterile saline coolant (Fig. 1a). Cementum was removed using Gracey curettes and a chisel. Reference notches were made with a #1 round bur on the root surface at the cementoenamel junction (CEJ), and on the crown surface, to indicate the precise center plane of the intrabony defects and to allow an optimal histomorphometric analysis. To prevent spontaneous healing and induce plaque accumulation, metal strips were fixed to the tooth surface in the intrabony defects with a self-cure dental adhesive resin cement (Super Bond C&B, Sun Medical Co., Ltd., Moriyama, Japan) (Fig. 1b). The flaps were repositioned and stabilized with 4–0 silk sutures. Ketoprofen for analgesia (Capisten IM 50 mg, 2 mg/kg, 0.1 ml/kg; Kissei Pharmaceutical Co. Ltd, Matsumoto, Japan) and an antibiotic (Mycillin Sol) were administered daily for 2 days following the surgeries. Intraoral periapical radiographs at selected sites including the teeth (P2 & P4) were taken immediately after the treatment. The sutures were removed after 14 days of healing. To promote plaque formation, the animals were fed a soft diet during the induction period (Fig. 1c). After 4 weeks, bone loss progression was confirmed by the radiographs and the metal strips were removed without flap reflection. Acrylic stents with a groove on the mid-proximal root surfaces where the deepest pockets were detected were then fabricated to standardize the location of periodontal probe for clinical measurements (Fig. 1d) during this study.

### Non-surgical periodontal therapy

**Fig. 2:**
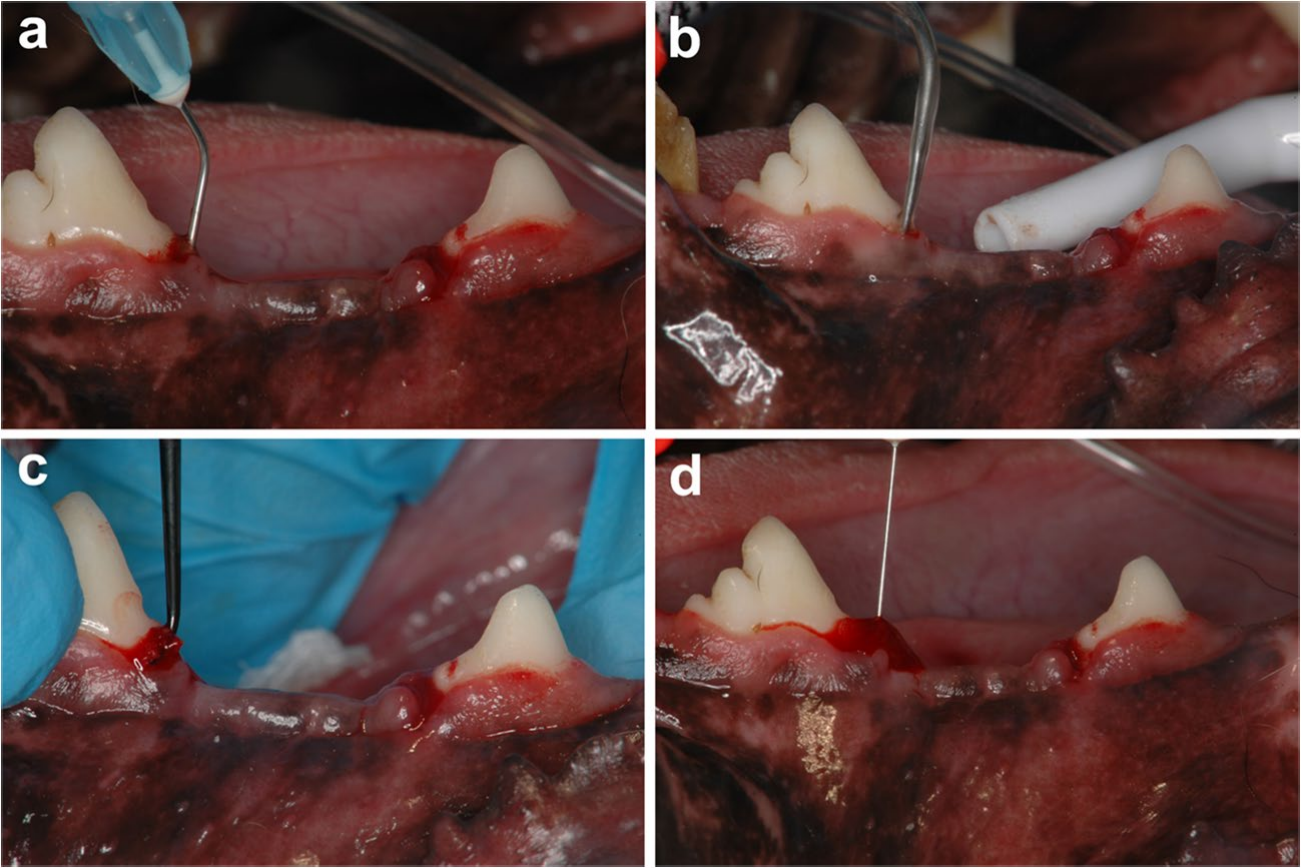
**a** Application of sodium hypochlorite/amino acid gel to the periodontal pocket. SRP performed using an ultrasonic device with an ultrasonic tip (**b**) and hand instruments (**c**). **d** Application of a xHyA gel to the periodontal pocket

Plaque control was performed with routine (3 times a week) flushing of the oral cavity with 2% chlorhexidine gluconate solution for 2 weeks prior to the treatment. To prevent mixing of gel type agents to the other site in the same side of the mandible, split-mouth design was employed in this study. Experimental 16 teeth (i.e., bilateral P2 & P4) were designated test and control side by coin flipping. Immediately before SRP, professional supragingival mechanical tooth cleaning was performed for the teeth. On one side, teeth were treated by SRP with sodium hypochlorite/amino acid gel followed by a cross-linked hyaluronic acid (xHyA) gel application (test group), whereas teeth of the contralateral side were treated by SRP only (control group) by the same experienced operator (T.N.). In the test group, SRP was performed as follows: in the teeth a sodium hypochlorite/amino acid gel (Perisolv®, an alkaline 0.95% sodium hypochlorite solution and a slightly viscous alkaline gel containing glutamic acid, leucine, lysine, carboxymethyl cellulose and titanium dioxide, Regedent AG, Zürich, Switzerland) was instilled into the periodontal pockets using a blunt needle for 30 s (Fig. 2a) prior to saline irrigation and instrumentation using an ultrasonic device (ENAC 10WA, Osada, Tokyo, Japan) and an ultrasonic tip (ST35, Osada, Tokyo, Japan) for 15 s (Fig. 2b), followed by hand instrumentation with manual curettes (LM Sharp Diamond Mini Gracey 11/12, 13/14 SD curettes, LM Dental™, Finland) through 5 traction movements in buccal and interproximal area (Fig. 2c), and the same process was repeated again. Following the final saline irrigation, the xHyA (hyadent BG®, a gel formulation containing butanediol diglycidyl ether-cross-linked HA (1000 kDA HA monomers) and non-cross-linked HA (2500 kDA) in a ratio 8:1, made from biotechnologically produced synthetic HA, REGEDENT AG, Zurich, Switzerland) gel (0.1 ml/tooth) was instilled in the pockets using a blunt needle (Fig. 2d). Teeth in the control group underwent the identical procedure except the sodium hypochlorite/amino acid and xHyA application. After the treatments, no antibiotics or analgesics were administrated, and the animals were fed a hard diet and the aforementioned oral hygiene regimen was performed daily for 8 weeks to reduce plaque formation.

### Clinical evaluation

The following clinical parameters were assessed using a periodontal probe (UNC 15 Hu-Friedy, Chicago, IL., USA) to the nearest mm on all teeth at one site per tooth by one experienced and blinded examiner (F.S.) at baseline (Fig. 1d) and 6 weeks following the treatment: (a) probing pocket depth (PPD), (b) clinical attachment level (CAL) measured from the acrylic stent margin to the bottom of the probed pocket and (c) bleeding on probing (BOP). BOP was evaluated simultaneously with PPD by recording the presence ( +) or absence (-) of bleeding up to 15 s after probing.

### Histologic preparation

Eight weeks after the non-surgical therapy, intraoral radiographs were taken, and the animals were euthanized with an overdose of sodium thiopental. The teeth were removed together with the surrounding soft and hard tissues. The tissue blocks were fixed in 10% buffered formalin, trimmed according to intraoral radiographs and the reference notch on the crown, and rinsed in phosphate-buffered saline. The samples were decalcified in Kalkitox™ (Wako Pure Chemical Industries, Ltd., Osaka, Japan), dehydrated, and embedded in paraffin. Serial 6-µm-thick sections were then prepared along the mesiodistal plane and were stained with hematoxylin and eosin or with azan.

### Histomorphometric analysis

All specimens were analyzed under a light microscope (BX51; Olympus Corp., Tokyo, Japan) equipped with a computerized image system (WinROOF2015; Mitani Corporation, Tokyo, Japan). For histomorphometric analysis, three sections approximately 90 µm apart were selected from the most central area of each two-wall defect, identified by the length of the root canal and the reference notches. The mean value of each histomorphometric parameter was then calculated for each site. To evaluate intra-examiner reproducibility, sixteen sections from all sites were read by a single blinded examiner at two different moments (48 h apart), and inter-calibration of the examiner was accepted at 90% level. The following parameters were measured by the examiner (T.I.). 1. Defect height (DH): distance between the apical extent of root planing and the CEJ. 2. Junctional epithelium length (JE): distance between the apical extension of the junctional epithelium and the CEJ. 3. Connective tissue adhesion (without cementum) (CT): distance between apical extent of the junctional epithelium and the coronal extent of the newly formed cementum. 4. New bone length (NB): distance between the apical extent of root planing and the coronal extent of newly formed alveolar bone along the root surface. 5. New bone area (NBA): newly formed trabecular bone within a template (5 × 5 mm) that served as a standardized proxy for the defect site. The template was aligned parallel to the root surface interfacing the apical extension of the root planing [45]. 6. New cementum length (NC): distance between apical extent of root planing and coronal extent of newly formed cementum on the denuded root surface.

7. New attachment length (NA): linear length of the root surface covered by NC adjacent to newly formed bone, with functionally oriented collagen fibers.

8. Periodontal ligament score (PDL score): which was obtained by grading the periodontal ligament with the reported scoring system outlined by Wikesjö et al. [46].

### Statistical analysis

The primary outcome of this study was the histomorphometric outcome in terms of NA, measured for the treatment groups at 8 weeks. Clinical parameters were evaluated as secondary outcomes. However, due to the limited number of pre-clinical studies in dogs with a comparative design and primary outcome, no power analysis for sample size calculation could be performed. For obvious ethical reasons, sample size was set to an absolute minimum (4 animals) and the animal was chosen as the unit for the statistical analysis. The means and standard deviations for each parameter were calculated for each of the treatment groups. Wilcoxon signed rank test was used to compare the clinical parameters between baseline and at the 6 week follow up. Mann–Whitney U test was used to compare the clinical and histological values between the control and test groups.

For the comparison of the proportions of sites showing bleeding on probing (BOP), Fisher's exact test was used. A *P* value of < 0.05 was considered statistically significant. All calculations were performed with statistical software (BellCurve for Excel; Social Survey Research Information Co., Ltd., Tokyo, Japan).

## Results

### Clinical observations

**Fig. 3:**
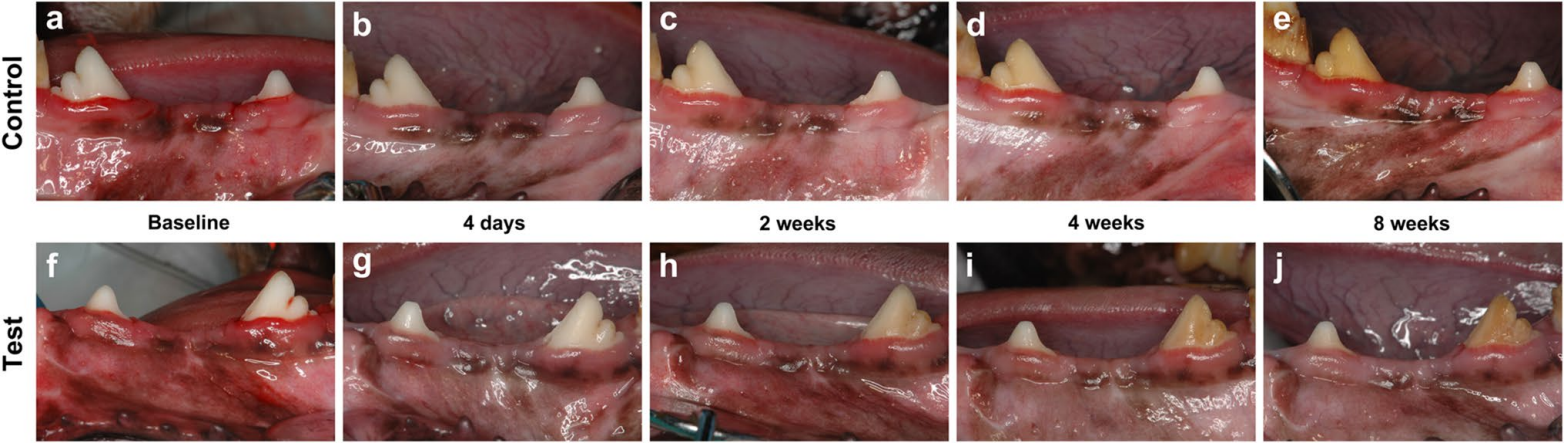
Clinical overview at baseline, 4 days, 2, 4 and 8 weeks in the control (**a**-**e**) and the test group (**f**-**j**)

Postoperative clinical healing was uneventful at all 16 (8 sites/group) sites in the control and test groups. No visible adverse reactions, including suppuration, abscess formation, or increased tooth mobility, were observed throughout the entire experimental period. Visual gingival redness seemed to remain longer or recur in the control group after the treatment (Fig. 3).

### Clinical measurements

The values for clinical parameters at the baseline and 6-week examinations in both treatment groups are shown in Table 1. The baseline examination revealed that the two study groups demonstrated similar characteristics for PPD, CAL, and BOP score with no significant differences between the groups. Both treatment groups showed clinical improvements at 6 weeks compared to baseline. Mean PPD reduction between the baseline and 6 weeks follow-up was statistically significantly different between the groups in favor for the test group (*p* < 0.05). The test group showed better results in terms of mean CAL gain compared to control group, however, no statistically significant difference was detected between the groups. The number of bleeding (BOP +) sites was markedly reduced in the test group at 6 weeks compared to baseline. Similarly, there was statistically significant difference in the score between the groups.

**Table 1:** Clinical parameters for each treatment at baseline and 6 weeks (means ± SD)

		*N* = 4 animals
Parameters	Control	Test
PPD (mm)		
Baseline	5.46 ± 0.82	5.50 ± 0.57
6 weeks	2.12 ± 0.32	1.25 ± 0.50
PPDreduction	3.34 ± 0.54	4.25 ± 0.50^†^
CAL (mm)		
Baseline	5.71 ± 0.84	5.59 ± 0.47
6 weeks	2.93 ± 0.23	1.68 ± 0.89
CAL gain	2.78 ± 0.79	3.90 ± 0.82
BOP ( +) n (%)		
Baseline	8 (100)	8 (100)
6 weeks	6 (75.0)	1 (12.5)^∗^^†^

*PPD* probing pocket depth, *CAL* clinical attachment level, *BOP* bleeding on probing

^∗^ Significantly different from baseline within each group (*p*<0.01)

^†^Significantly different from control group (*p*<0.05)

### Descriptive histology

**Fig. 4:**
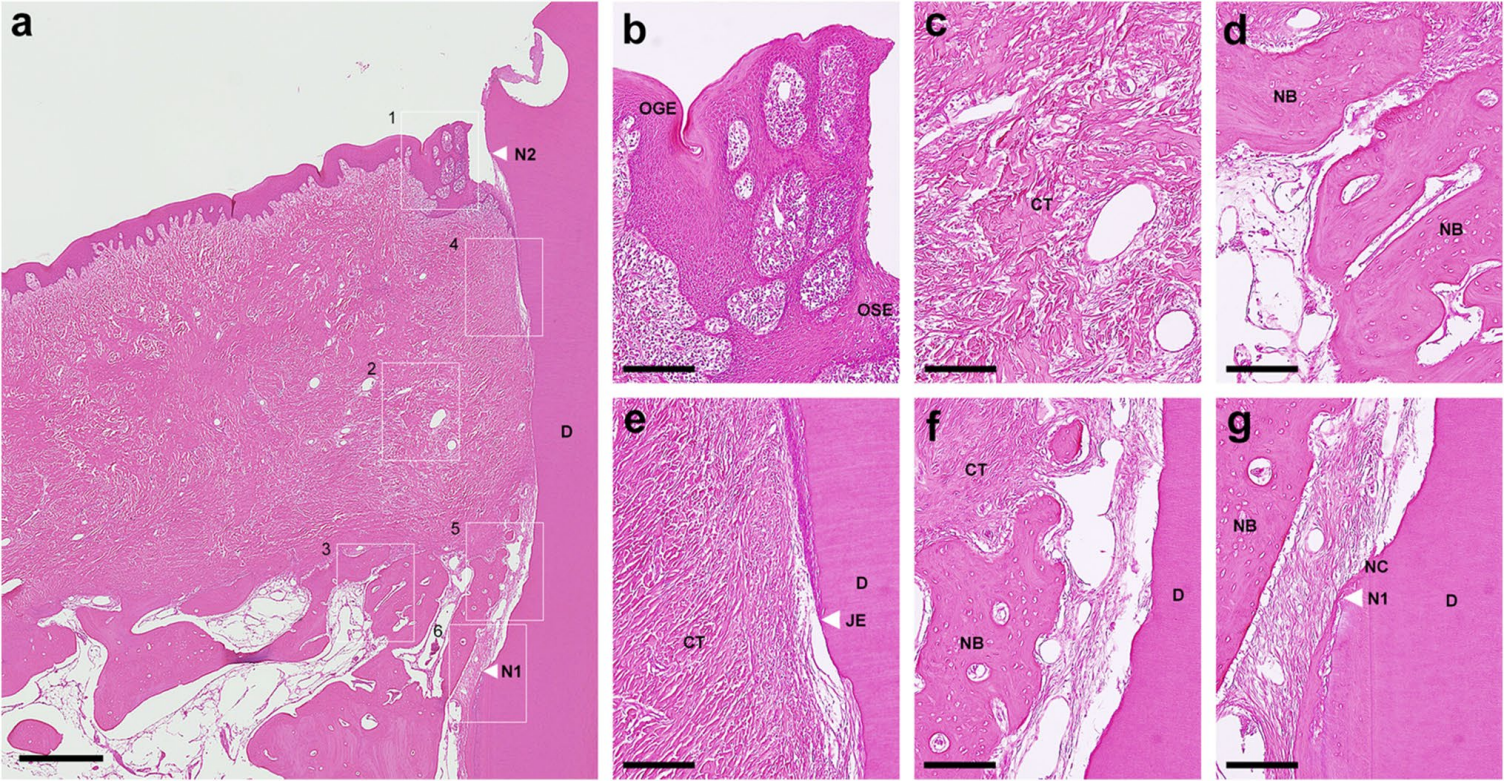
**a** Histologic overview of defect treated with SRP alone (control group). (scale bar, 1 mm; hematoxylin and eosin stain). **b** Higher magnification of the box 1 area. **c** Higher magnification of the box 2 area. **d** Higher magnification of the box 3 area. **e** Higher magnification of the box 4 area. **f** Higher magnification of the box 5 area. **g** Higher magnification of the box 6 area. (scale bar, 200 µm; hematoxylin and eosin stain). D, root dentin; N_1,_ apical end of root planing; N_2,_ cementoenamel junction; OGE, oral gingival epithelium; OSE, oral sulcular epithelium; JE, apical end of junctional epithelium; CT, gingival connective tissue; NB, new bone; NC, new cementum

In the control group, a collapse of the soft tissue could be observed in three teeth and the oral gingival epithelium was partially thickened with deeper rete ridges than normal one (Fig. 4a and b) in four teeth. Also, most sites (seven out of eight teeth) showed the slight to moderate widespread inflammatory cell infiltrate mostly at the tips of gingiva (Fig. 4a and b). Downgrowth of junctional epithelium was detected slightly below the CEJ (Fig. 4a and e). Periodontal defect was mostly occupied by fibrous connective tissue (Fig. 4a and c) and slight superficial (two teeth) and inflammatory (one tooth) root resorption areas were seen on the root surfaces without cementum formation (Fig. 4e and f) in three teeth. Extensive proximal host bone resorption and varying degrees of spontaneous bone formation occurred along the root surfaces (Fig. 4a and d) in all sites. Two teeth in the control group presented no cementum formation at all and predominantly acellular cementum formation was restricted at the apical extension of instrumentation (Fig. 4a, f and g) in five teeth. Most specimens (5/8, 62.5%) in the control group showed non-functional disordered periodontal ligament like tissue or collagen fibers detached from the root surfaces (Figs. 4f and g and Fig. 6a).

**Fig. 5:**
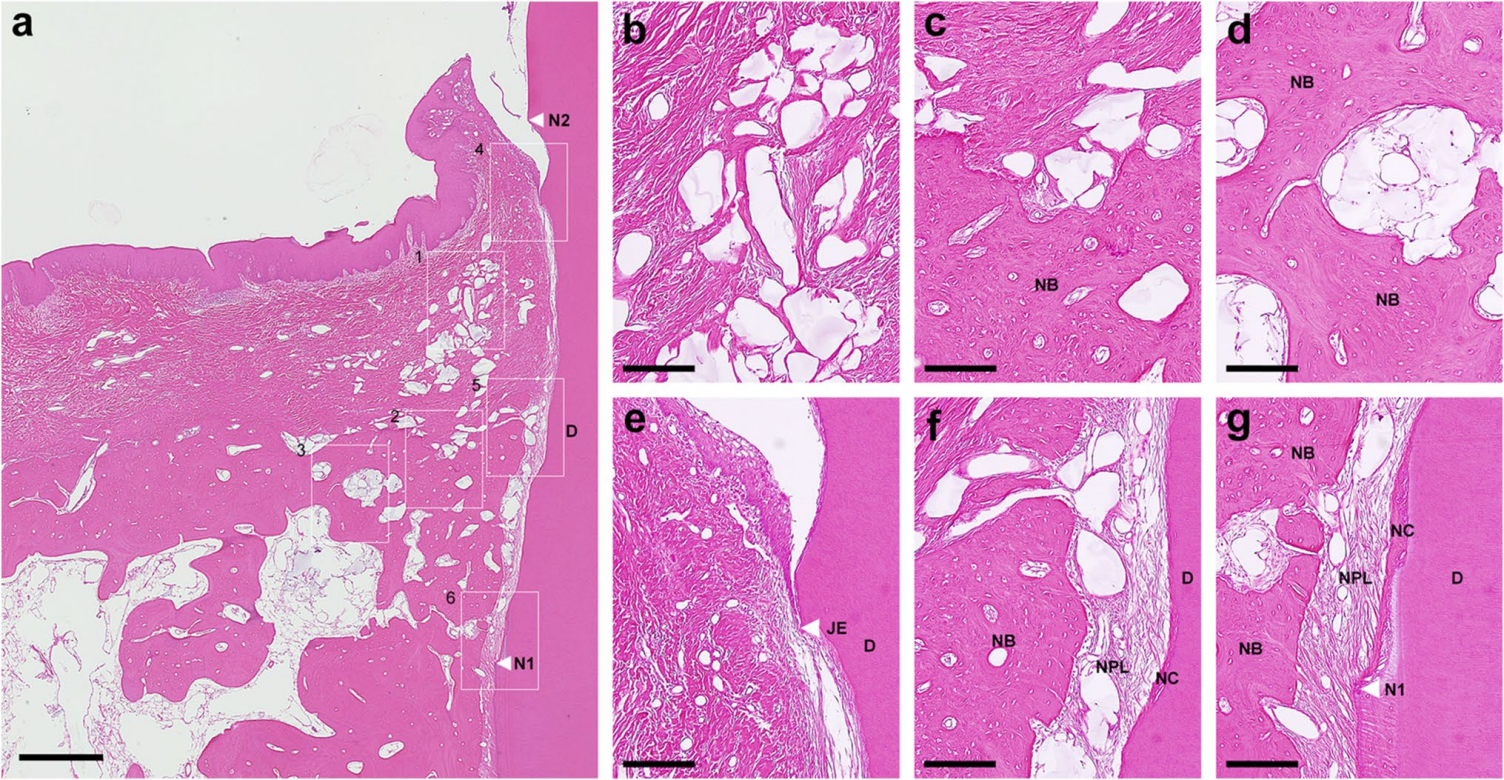
**a** Histologic overview of defect treated with SRP with a sodium hypochlorite and amino acids gel and a cross-linked hyaluronic acid gel (xHyA) gel (test group). (scale bar, 1 mm; hematoxylin and eosin stain). **b** Higher magnification of the box 1 area. **c** Higher magnification of the box 2 area. **d** Higher magnification of the box 3 area. **e** Higher magnification of the box 4 area. **f** Higher magnification of the box 5 area. **g** Higher magnification of the box 6 area. (scale bar, 200 µm; hematoxylin and eosin stain). D, root dentin; N_1,_ apical end of root planing; N_2,_ cementoenamel junction; JE, apical end of junctional epithelium; CT, gingival connective tissue; NB, new bone; NC, new cementum; NPL, new periodontal ligament

In the test group, residual xHyA with reticular appearance was well integrated with gingival connective tissue at the coronal portion of the defects in all sites (Fig. 5a and b). Some remnants of xHyA were observed around/in the newly formed bone and occasionally between new cementum and new bone (Fig. 5a, c, d and f). Marked soft tissue atrophy or pathological change of gingival epithelium was not noted (Fig. 5a). A limited inflammatory response was observed in the tips of gingiva around three teeth (Fig. 5e). Apical extension of junctional epithelium was mostly restrained at the CEJ (Fig. 5a and e). Superficial root resorption was detected on the root surface of one tooth. New bone formation extended from the host bone toward the coronal region of the defects (6/8, 75%) (Fig. 5a). Newly formed bone was well integrated with the original bone and characterized by cancellous bone, which consists of a network of bony trabeculae containing bone marrow, blood vessels, osteoblasts, and osteocytes (Fig. 5a, c and d). A continuous layer of new cellular/acellular cementum was seen, with or without inserting collagen fibers running perpendicular to the root surfaces, was observed covering half of the defect area (Fig. 5a, f and g) in 5 teeth. The highly vascularized and dense new periodontal ligament-like tissue, which was formed between the new cementum and new bone (Figs. 5f and gandFig. 6b), maintained its width up to the coronal portion in the test group. No ankylosis was observed in any of the teeth.

**Fig. 6:**
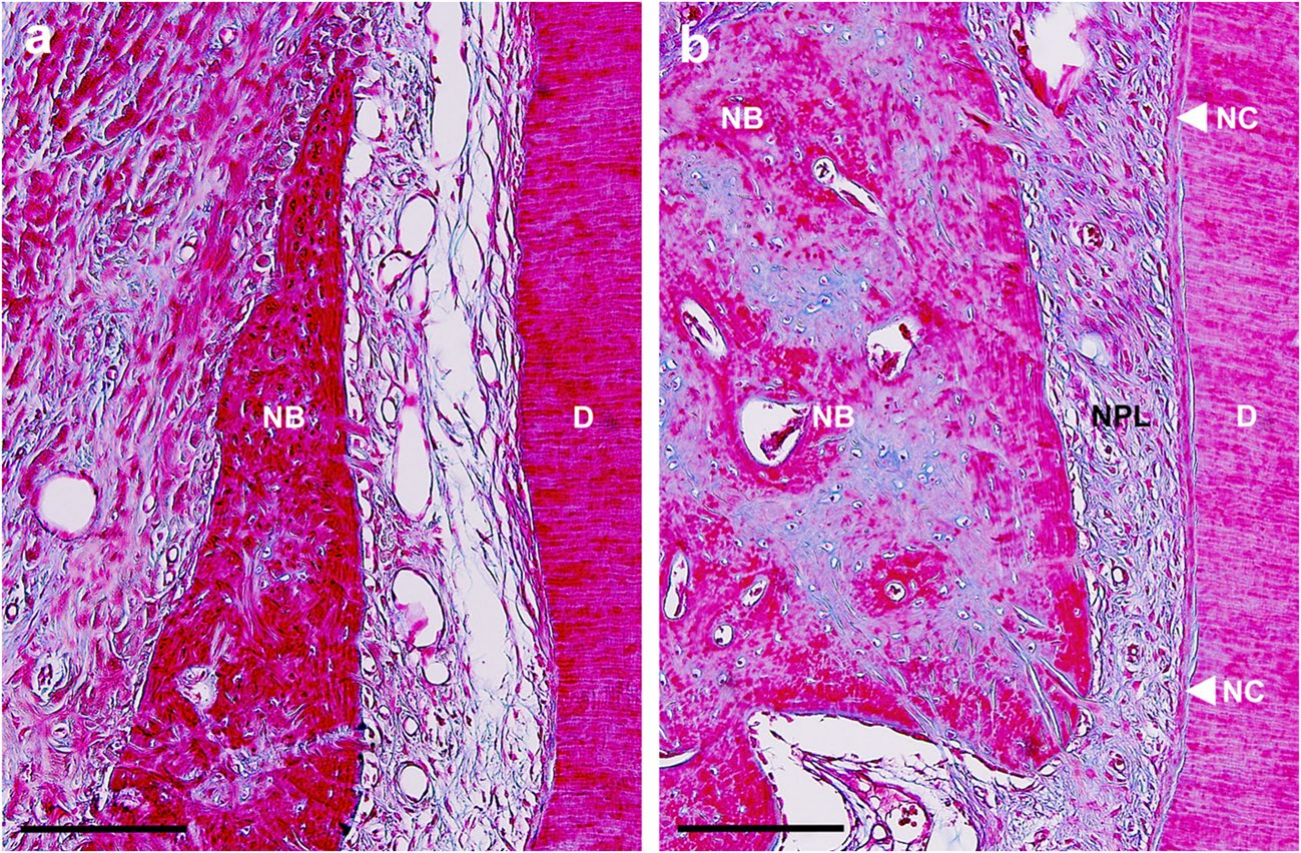
**a** Higher magnification view of the middle portion of the defect treated with SRP alone (control group). Loosely arranged collagen fibers were seen near the root dentin without new cementum. (scale bar, 200 µm; azan stain). **b** Higher magnification view of the middle portion of the defect treated with SRP with a sodium hypochlorite and amino acids gel and a cross-linked hyaluronic acid gel (xHyA) gel (test group). Dense obliquely oriented collagen fibers were observed between the new bone and cementum. (scale bar, 200 µm; azan stain). D, root dentin; NB, new bone; NC, new cementum; NPL, new periodontal ligament

### Histomorphometric analysis

The results of histomorphometric analysis are shown in Table 2. No statistically significant differences were detected between the groups in regard to the following measurements (DH, JE, and CT). However, the length of CT (without cementum formation) in the test group was smaller than that observed in the control group. The length of new cementum was statistically significantly (*P* < 0.01) greater in the test (2.46 ± 0.77 mm) group than in the control (0.85 ± 0.84 mm) group. The test (1.75 ± 0.65 mm) group yielded statistically significantly (*P* < 0.05) greater formation of new attachment (i.e., linear length of NC adjacent to newly formed bone, with functionally oriented collagen fibers) compared with control (0.48 ± 0.79 mm) group. Moreover, the PDL scores in the test (2.87 ± 1.59) group was statistically significantly (*P* < 0.05) higher than that in the control (1.00 ± 0.94) group. The amount of newly formed bone (e.g., the length of NB and the area of NB) in the test (3.01 ± 0.64 mm and 5.75 ± 2.21 mm^2^, respectively) group was statistically significantly (*P* < 0.05) greater than that in the control (2.26 ± 0.64 mm and 3.14 ± 1.94 mm^2^, respectively) group.

**Table 2:** Histomorphometric comparisons between test and control groups 8 weeks after treatment. (means ± SD)

		*N* = 4 animals
Parameters	Control	Test
DH (mm)	5.60 ± 0.42	5.77 ± 0.52
JE (mm)	1.22 ± 0.45	1.03 ± 0.31
CT (mm)	2.84 ± 1.33	1.86 ± 1.00
NC (mm)	0.85 ± 0.84	2.46 ± 0.77^∗∗^
NA (mm)	0.48 ± 0.79	1.75 ± 0.65^∗^
PDL score (1–5)	1.00 ± 0.94	2.87 ± 1.59^∗^
NB (mm)	2.26 ± 0.64	3.01 ± 0.64^∗^
NBA (mm^2^)	3.14 ± 1.94	5.75 ± 2.21^∗^

*DH* defect height, *JE* junctional epithelium length, *CT* connective tissue adhesion (without cementum), *NB* new bone length, *NBA* new bone area, *NC* new cementum length, *NA* new attachment length, *PDL score* periodontal ligament score

^∗^ Significantly different from control group (*p* < 0.05)

^∗∗^ Significantly different from control group (*p* < 0.01)

## Discussion

The present study has, for the first time, provided histological evidence of periodontal regeneration following the adjunctive subgingival application of sodium hypochlorite/amino acids and a xHyA gels to SRP. The histological results were consistent with the greater clinical improvements observed in terms of PPD reduction, CAL gain, and reduction of inflammation in the test group compared to the control group, which in tern, underscores the potential clinical significance of these findings.

The clinical findings obtained in this animal study are in line with the results from clinical studies reporting that SRP combined with sodium hypochlorite/amino acid and xHyA gels resulted in statistically significantly higher clinical improvements evidenced through PPD reduction, CAL gain, and decrease of BOP score (values) as compared to baseline [42, 43] or SRP alone [44]. In line with the clinical findings, the histologic analysis revealed that the test treatment yielded statistically significantly greater amounts of new connective attachment and new cementum formation than the control one. In the defects treated by SRP with a sodium hypochlorite and amino acids containing gel and a xHyA gel, dense functionally oriented collagen fibers with numerous blood vessels were predominantly observed between the newly formed cementum and the newly formed bone showing high PDL scores compared to the teeth treated by SRP alone. In addition, statistically significantly greater new bone was measured in the test group compared with the control one.

When interpreting these positive results, it must be emphasized that the present study has used the combination of the two different materials as a single treatment adjunctive to SRP. The cleaning effect was expected by the sodium hypochlorite and amino acids gel, which create chloramines. Chloramines have a strong antimicrobial effect and minimize the effects of hypochlorite on sound dentin/root cementum and healthy soft tissue [26, 28, 47]. Additionally, in vitro, and clinical studies have demonstrated that the sodium hypochlorite/amino acid gel can facilitate SRP to disrupt the biofilm, by dissolving necrotic tissue, and by softening calculus and thus reducing friction during instrumentation [26–29]. The positive effect on the healing was expected by subsequent application of a xHyA gel since several in vitro studies have demonstrated that HyA significantly stimulates blood clot formation [30, 48], induces angiogenesis [30, 33] and increases osteogenesis [30, 34] as a biological modulator for promoting periodontal wound healing/regeneration. Additionally, recent studies have shown that the surgical application of the same high molecular xHyA yielded statistically significant improvements characterized by PPD reduction and CAL gain in human intrabony defects [38] and effectively promoted periodontal tissue regeneration in canine 2-wall intrabony, gingival recession and class III furcation defects [39–41].

When interpreting the results it is important to point out that the study did not include treatment groups treated with sodium hypochlorite/amino acids gel or xHyA. Therefore, it is unclear to what extent each of the used adjunctive materials may have contributed to the favorable outcomes obtained in the test group. A very recent animal study has shown that treatment of experimental periodontitis in rats using the combination of sodium hypochlorite/amino acids gel and SRP yielded to better outcomes in terms of gingival bleeding index, tooth mobility and the overall aspect of the gingival structures than treatment with SRP alone. However, the histologic evaluation demonstrated a predominantly reparative type of healing characterized by non-functionally oriented collagen fibers and unrestored alveolar ridges following the treatment with sodium hypochlorite/amino acids gel and SRP [49]. Megally et al. reported that subgingival ultrasonic debridement with sodium hypochlorite/amino acid gel resulted in a clinically relevant PPD reduction and CAL gain in residual pockets in subjects in maintenance care. However, these improvements were comparable to that obtained in the ultrasonic debridement alone without statistically significant differences [28]. Also, Pilloni et al. demonstrated that subgingival instrumentation with the local adjunctive use of xHyA yielded statistically significant clinical and microbiological improvements compared to baseline in residual periodontal pockets, although there was a lack of statistically significant differences in the outcomes following the subgingival instrumentation with placebo control [50]. These results may justify the rationale for the novel approach including the combined use of the two materials adjunctive to SRP as a single treatment procedure. Additionally, it is important to emphasize that the present animal study was designed to assess the biologic potential of the very recently introduced clinical protocol for nonsurgical periodontal treatment [42–44].

An interesting observation in this study is related to the changes in bleeding scores; i.e., while gingival inflammation decreased within a week in both control and test groups, gingival swelling and redness gradually increased during the 8 weeks following treatment in the control group. On the contrary, no such increase occurred in the test group. This clinical observation was consistent with the histologically observed inflammatory cell infiltrate at the coronal part of gingiva in the control group. In the test group, varying degrees of xHyA remnants were consistently observed, but they did not appear to interfere with tissue integration in all periodontal defects around teeth. These findings are also in agreement with those from previous preclinical studies that have demonstrated the presence of residual xHyA in two-wall intabony and class III furcation defects in dogs [40, 41].

An important finding that warrants further attention is the fact that this novel approach for non-surgical periodontal therapy (NSPT) resulted in chronic two-wall intrabony defects in comparable amounts of NC (2.46 ± 0.77 mm) and NA (1.75 ± 0.65 mm) to those (3.20 ± 1.29 mm, 2.43 ± 1.29 mm respectively) obtained in the same initial size of acute two-wall intrabony defects treated with a surgical approach and the application of xHyA in dogs [40]. These findings indicate that the application of xHyA in NSPT is clinically beneficial, and the high molecular weight xHyA can maintain its stability for 4 to 8 weeks [40, 41, 51]. From a clinical perspective, the results of the present study suggest that this novel approach offer additional benefits in NSPT and decrease the need for surgical periodontal therapy.

In contemporary periodontology, the adjunctive use of EMD [20, 21], antibiotics [22, 23], lasers and antimicrobial photodynamic therapy [24, 25], has been repeatedly investigated as adjunctive approaches to NSPT. However, additional benefits compared to SRP alone were not consistently observed while the relatively high cost, some risks of allergy and the necessity of specific equipment for these treatment modalities need also to be considered. On the other hand, the novel approach evaluated in the present study is based on the use of a highly biocompatible and non-animal origin materials adjunctive to SRP. The sodium hypochlorite cleaning gel may offer further advantages to NSPT by facilitating the mechanical removal of the biofilm, thus enhancing the effects of the xHyA gel [42]. Moreover, the xHyA gel enhances blood clot stability and attracts several growth factors [38, 52, 53] which play a key role in periodontal wound healing/regeneration [54]. However, it needs to be emphasized that despite the fact that these results are encouraging, they were obtained in experimentally created defects including a small number of animals. Therefore, further studies are required to confirm the clinical relevance and the predictability of this novel treatment approach in NSPT.

## Conclusion

In conclusion, the present data offer histological evidence supporting the use of adjunctive subgingival application of sodium hypochlorite/amino acid and xHyA gels in NSPT to enhance periodontal wound healing and regeneration.

## Data Availability

The data that support the findings of this study are available from the corresponding author upon reasonable request.
